# Computational biotechnology guides elucidation of the biosynthesis of the plant anticancer drug camptothecin

**DOI:** 10.1016/j.csbj.2021.06.028

**Published:** 2021-06-22

**Authors:** Emily Amor Stander, Thomas Dugé de Bernonville, Nicolas Papon, Vincent Courdavault

**Affiliations:** a Université de Tours, EA2106 “Biomolécules et Biotechnologies Végétales”, Tours, France; b Univ Angers, Univ Brest, GEIHP, SFR ICAT, Angers, France

**Keywords:** Medicinal plants, Next-generation sequencing, Genome mining, Pharmaceuticals, Biosynthetic pathway, Synthetic biology

## Abstract

Camptothecin is a clinically important monoterpene indole alkaloid (MIAs) used for treating various cancers. Currently, the production of this biopharmaceutical hinges on its extraction from camptothecin-producing plants, leading to high market prices and supply bottlenecks. While synthetic biology combined with metabolic approaches could represent an attractive alternative approach to manufacturing, it requires firstly a complete biosynthetic pathway elucidation, which is, unfortunately, severely missing in species naturally accumulating camptothecin. This knowledge gap can be attributed to the lack of high-quality genomic resources of medicinal plant species. In such a perspective, Yamazaki and colleagues produced the first described and experimentally validated chromosome-level plant genome assembly of *Ophiorrhiza pumila*, a prominent source plant of camptothecin for the pharmaceutical industry. More specifically, they have developed a method incorporating Illumina reads, PacBio single-molecule reads, optical mapping and Hi-C sequencing, followed by the experimental validation of contig orientation within scaffolds, using fluorescence *in situ* hybridization (FISH) analysis. This relevant strategy resulted in the most contiguous and complete *de novo* plant reference genome described to date, which can streamline the sequencing of new plant genomes. Further mining approaches, including integrative omics analysis, phylogenetics, gene cluster evaluation and comparative genomics were successfully used to puzzle out the evolutionary origins of MIA metabolism and revealed a short-list of high confidence MIA biosynthetic genes for functional validation.

## Graphical abstract

**Figure:**
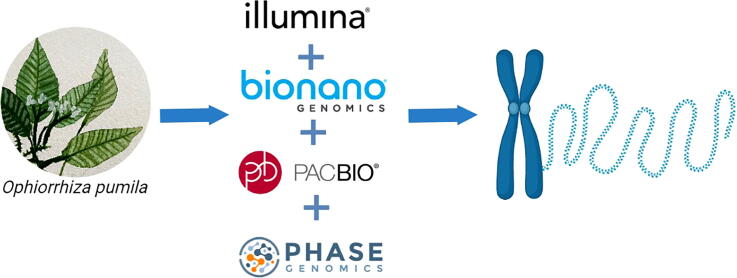

Cancer is overtaking cardiovascular disease as the leading cause of death worldwide [1]. Many of the modern-day anticancer drugs used for treating a variety of cancers are extracted or derived from plant monoterpene indole alkaloids (MIAs) including camptothecin, vinblastine and vincristine [2]. MIAs correspond to a diverse group of plant specialized (or secondary) metabolites produced in species related to the Gentianales (Apocynaceae, Rubiaceae and Loganiaceae) and Cornales (Nyssaceae). The astounding chemodiversity found within these species stems from a single common precursor, strictosidine, which is formed by a condensation reaction between the iridoid monoterpene, secologanin, and tryptamine, produced from the decarboxylation of tryptophan (Fig. 1, [3]). Although some pharmaceutical MIAs can be synthesized chemically at a laboratory scale, most clinically important MIAs display complex structures, which prevent commercial-scale production. Therefore, many of these drugs or drug precursors are still extracted from plant resources which significantly increase the market price and in turn decrease the supply potential due to low *in planta* production and challenging extraction techniques.

**Fig. 1:**
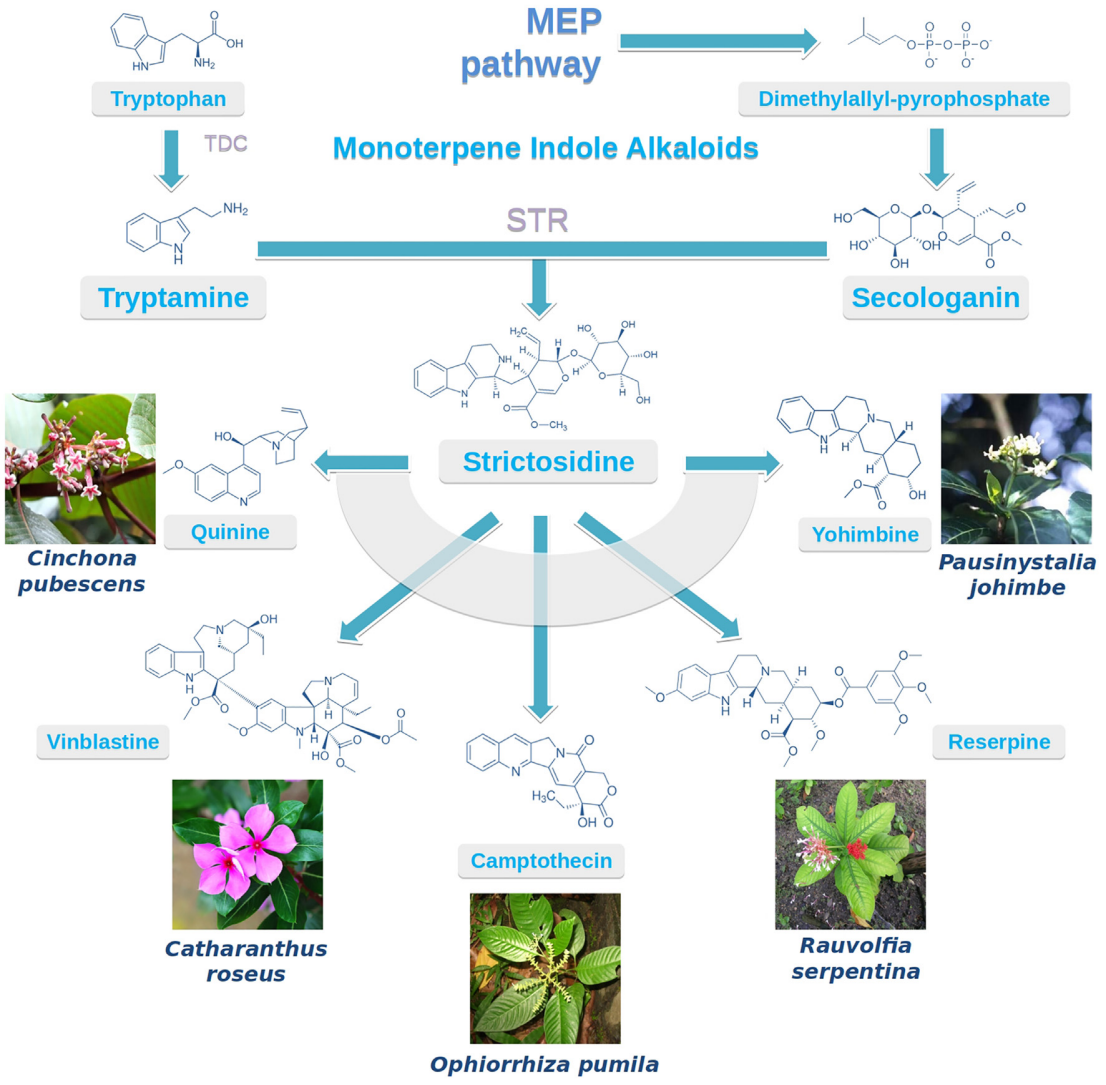
The astounding assortment of MIAs found in some prominent medicinal plants. MIAs are mainly derived from a single common intermediate, strictosidine, which is the product of the condensation reaction between tryptamine (derived from the decarboxylation of tryptophan by TDC) and secologanin (derived from the iridoid pathway). TDC: tryptophan decarboxylase, STR: strictosidine synthase.

In this context, recent advancement in synthetic biology has provided attractive alternatives for producing these biopharmaceuticals at affordable prices. However, these approaches require in-depth knowledge on the biosynthetic pathways of these molecules, *in planta*, which obviously primarily relies on the availability of high-quality genomic resources. To date, most of the vinca biosynthetic pathways have been elucidated in the Madagascar periwinkle *Catharanthus roseus* whilst knowledge over camptothecin-producing species remains limited. However, it is well entrenched in the literature that camptothecin producing species are spread across different families, including Rubiaceae (*Ophiorrhiza pumila*), Apocynaceae (*Chonemorpha fragrans*) and Nyssaceae (*Camptotheca* spp.). Unfortunately, the biosynthetic origin of camptothecin still remains a matter of debate, either from strictosidine as proposed in *O. pumila* [4] or from strictosidinic acid in *Camptotheca acuminata* [5].

Due to the increased accessibility and affordability of 2nd generation short-read sequencing, the number of available plant reference genomes is rapidly expanding. However, many of the resulting genome sequences are highly fragmented with assembly gaps, precipitating issues of orientation and ordering of contigs within scaffolds. This is mainly caused by the unique challenges that plant genomes introduce with their large genome sizes, heterozygosity, polyploidy and multiple repeat regions. Furthermore, most of the comparative genomic analyses used in biosynthetic pathway discovery include biosynthetic gene cluster- (BGC), synteny- and paleogenomics investigations, which heavily depend on high continuity input genome sequences and accurate gene order. Currently, specific library designs (mate-pairs or linked reads), 3rd generation long-read sequencing such as HiFi Pacbio reads and scaffolding technologies using optical maps or linked chromatin fragments (Hi-C) have significantly improved the alignment and ordering of contigs towards pseudomolecules through multistage scaffolding approaches [6]. However, it still requires hard work to decipher the orientation of these contigs within scaffolds and is usually prone to errors. Genetic maps can be used to correct scaffold anchoring but are not so common for non-model species.

In such a perspective, the recent paper by the Yamazaki group [4] not only re-emphasized the advantage of using a multi-ordered scaffolding approach for achieving more contiguous plant genome assemblies, but also demonstrates the importance of consistent *in silico* assembly validation following each scaffolding step using physical mapping technologies. However, the final experimental validation stage of the pipeline using fluorescence *in situ* hybridization (FISH) proved to be crucial in identifying an orientation error in the final *O. pumila* genome, pioneering an additional quality control check towards guaranteeing complete genomes.

By sequentially incorporating four state-of-the-art technologies including 2nd generation seq (Illumina), 3rd generation (Pacbio) and physical mapping methods (Bionano optical maps, Hi-C libraries), Rai and colleagues achieved a chromosome-level assembly of the *O. pumila* genome (Fig. 2). This task was initiated by assembling *de novo* PacBio reads using Canu [7]. The resulting Canu-assembly was subsequently used as a reference genome to guide the assembly of Bionano optical maps into a Bionano *de novo* assembly using the Bionano Solve software suite. The Canu-assembly consisting of 243 contigs was scaffolded using the Bionano *de novo* Optical assembly into a hybrid Canu-bionano assembly, consisting of 45 scaffolds (scaffold N50: 21 Mb) using Bionano Solve software. Hi-C proximity ligation reads were then mapped to the hybrid Canu-bionano assembly to further scaffold fragments. This sequential scaffolding method using Bionano optical maps, followed by Hi-C library scaffolding, significantly improved the continuity of the assembly from 45 to 13 scaffolds and reduced the number of gaps from 117 to 85. The continuity of the genome was further improved through gap filling and three rounds of polishing using PacBio reads, followed by error correction with Pilon using Illumina paired-end reads resulting in a final reference genome assembly of 21 gaps. The N50 value of this assembly (18.49 Mb) stands out as a significant improvement to previously published MIA producing plant genomes of *C. roseus* V2 (contig N50: 0.046 MB), *Gelsemium sempervirens* V3.0 (contig N50: 0.051 Mb) and *C. acuminata* (contig N50: 0.1 Mb). Most impressively, the authors were able to experimentally validate the accuracy of the genome using FISH, establishing an important benchmark for future plant genome assembly methodologies. In this case, the authors detected repeat-free non-homologous regions close to gaps and used them as probes to validate contig orientation. Synteny analysis between *O. pumila* and *Coffea canephora* (which is often used as the representative genome of the Rubiaceae family), highlighted a misassembly in the coffee genome which was subsequently experimentally confirmed with FISH analysis. This demonstrates how the near-complete *O. pumila* genome represents an outstanding standard resource insofar it can support validation and improvement of other plant genomes from underrepresented families. Finally, the authors were able to puzzle out the evolutionary origins of MIA metabolism by investigating the high-quality *O. pumila* genome with the latest genome mining approaches, including integrative omics analysis, phylogenetics, BGC evaluation and comparative genomics (Fig. 3).

**Fig. 2:**
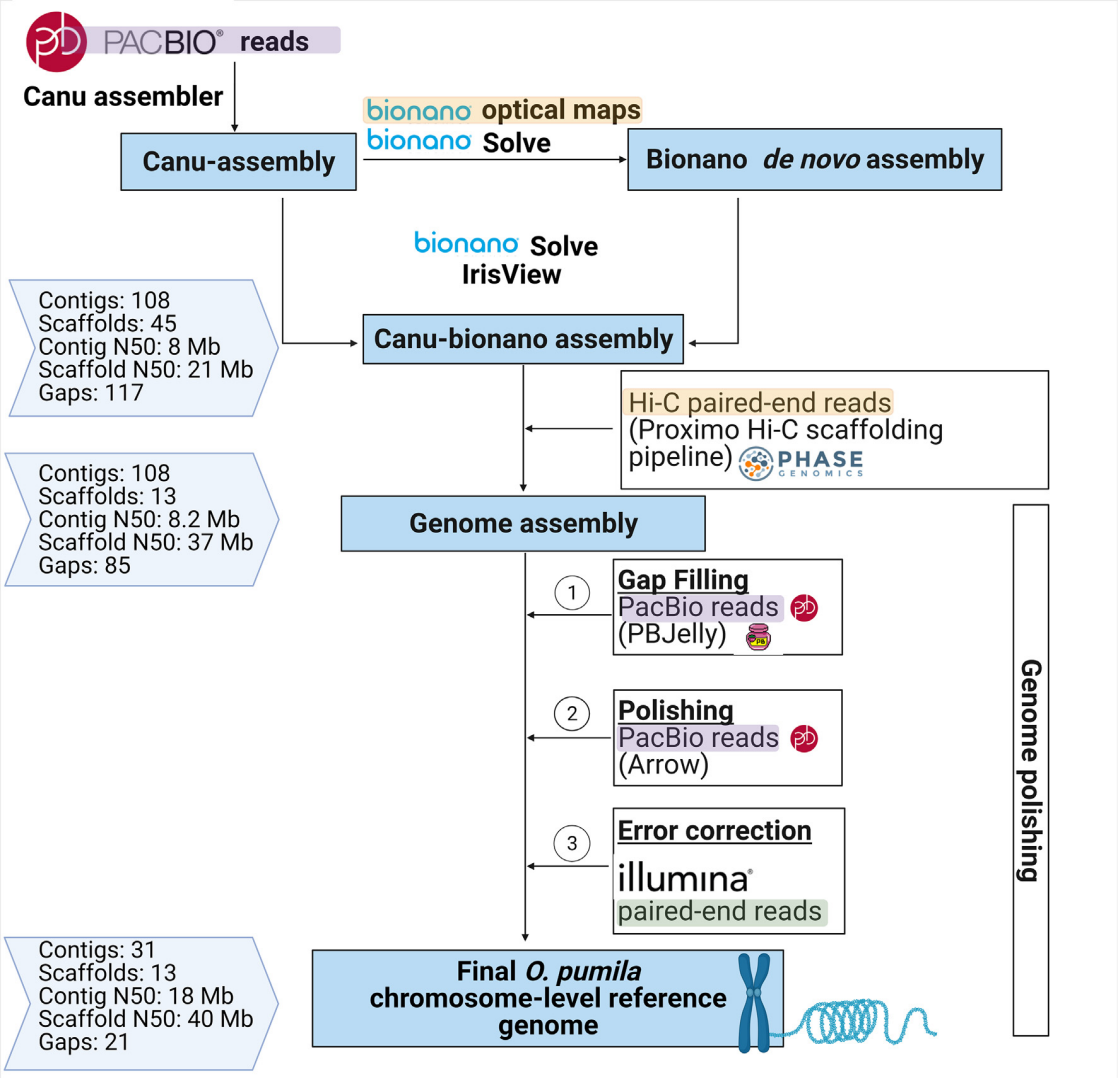
Sequential multistage genome scaffolding strategy. This combines 2nd generation (green), 3rd generation- (purple), and scaffolding technologies (orange), to generate the chromosome-level *O. pumila* reference genome assembly. (For interpretation of the references to colour in this figure legend, the reader is referred to the web version of this article.)

**Fig. 3:**
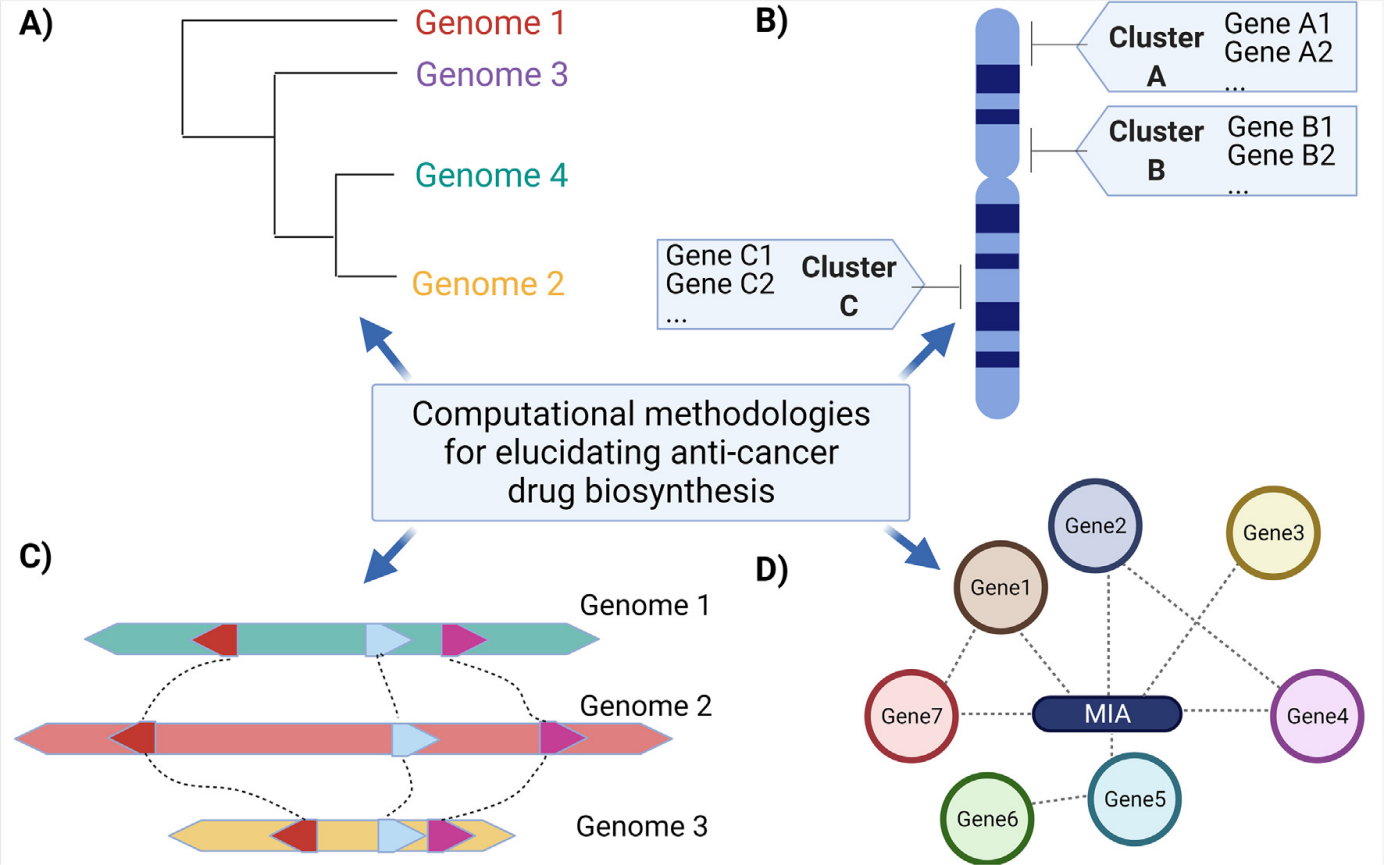
The biocomputational methodologies that were integrated into this study included. A) Phylogeny, B) Biosynthetic Gene Clusters, C) Synteny and collinearity analysis across different MIA-producing species, and D) gene-metabolite correlation networks.

The divergence times between *O. pumila* and *C. acuminata* (~120 mya), *C. roseus* (~68 mya) and coffee (~47 mya) were estimated thanks to a maximum likelihood phylogenetic tree that was constructed using single-copy genes assigned to orthologous gene families. A synonymous substitution per synonymous sites (Ks) analysis was able to detect a previously reported whole-genome duplication (WGD) event across all core eudicots, as well as an additional and unprecedented WGD event in *C. acuminata*. The similar biochemical profiles found across diverse orders of MIA-producing plant species may indicate that these species retained the MIA biosynthetic ability after WGD within core eudicots. In addition, the WGD event in *C. acuminata* further suggests that MIA biosynthesis was expanded after this event, catalyzing the alternative strictosidinic acid-derived camptothecin biosynthetic pathway. Overall, this example of convergent evolution in MIA biosynthesis highlights the importance of understanding MIA evolution across the plethora of MIA-producing species. This knowledge may in turn shed light on the uniqueness in pathways between species to guide gene identification within each species.

Next, the authors initiated a targeted approach to identify 1226 putative *O. pumila* MIA-related genes based on high sequence homology to 94 functionally validated MIA genes that were previously characterized in other plants. Sequence-based clustering of the putative candidates with the 94-gene set further reduced the number of candidate sequences to 216 genes, representing 40 functionally validated genes. The normalized expression profiles of this putative MIA gene set was found to be strongly correlated (PCC > 0.7) with MIA metabolite accumulation across *O. pumila* tissue types, further adding confidence to the annotated MIA-related functions of these genes. This high-confidence putative MIA gene set was subsequently used to search for MIA-specific BGCs throughout the *O. pumila* reference genome. Secondary metabolite BGCs have been well described in fungi [8], and are also found in some plants [9]. The availability of high-quality, continuous genomes is an important prerequisite for identifying BGC with computational tools. For example, the pathway of the anticancer compound noscapine was initially elucidated by identifying the 10-gene noscapine BGC in opium poppy (*Papaver somniferum*) using bacterial artificial chromosomes [10]. Once the genome of *P. somniferum* became available, five additional genes were identified within this genomic region, further elucidating the pathway towards the biosynthesis of thebaine- an intermediate of analgesic molecules morphine and codeine [11].

The PlantClusterFinder pipeline [12] was used to identify 33 MIA BGC across 8 of the 11 chromosomes in *O. pumila* [4]. Synteny between these BGCs from different MIA-producing species was found to be statistically significant based on one-sided Fisher’s exact test, highlighting once again the importance of these genomic regions for the expansion and evolution of MIA biosynthesis. Among the conserved BGC, a cluster containing a functionally characterized TDC and STR (Fig. 1) together with a multi-antimicrobial extrusion (MATE), a protein previously described in the genomes for MIA producing species *C. roseus* and *G. sempervirens* [13], was detected also in *O. pumila*. Within this cluster, Rai and colleagues hypothesize that the emergence and retention of STR (Fig. 1) after a whole-genome triplication event amongst core eudicots was the driving force behind MIA production in selected plant species. Indeed, while Ophiorrhiza and coffee are both phylogenetically related to the Rubiaceae family and share significant collinearity as established through synteny analysis, coffee appears to have lost a functional STR enzyme, explaining the lack of strictosidine and MIA production in this species. This observation definitively propels computational BGC identification as a concrete method for candidate gene selection, for functional characterization as well as for evolutionary studies.

In conclusion, the Yamazaki group has reiterated the advantage of using the sequential scaffolding strategy in *de novo* genome assemblies using orthogonal evidence derived from Bionano optical maps and Hi-C library sequencing. Although this strategy is by no means a silver bullet for all plant genomes, the authors do highlight the importance of the order in which these technologies are integrated for the resulting scaffold continuity and should therefore be optimized for each new genome assembly through combinatorial testing followed by continued assembly validation. The advantage of using a near-complete chromosome-level reference plant genome was further demonstrated in paleogenomics studies, providing an excellent resource that will strongly support future phytochemical genomics programs. This resulting resource opens up new perspectives on the camptothecin biosynthetic pathway, which in turn should promote metabolic engineering strategies to implement the sustainable- and cost-efficient industrial-scale production of this inescapable anti-cancer compound.

## Funding

We acknowledge funding from the ARD CVL Biopharmaceutical program of the Région Centre Val de Loire (ETOPOCentre project), EU Horizon 2020 research and innovation program (MIAMi project-grant agreement N°814645), and ANR MIACYC (ANR-20-CE43-0010).

## CRediT authorship contribution statement

**Emily Amor Stander:** Conceptualization, Writing - original draft, Writing - review & editing. **Thomas Dugé de Bernonville:** Conceptualization, Writing - original draft, Writing - review & editing. **Nicolas Papon:** Conceptualization, Supervision, Writing - original draft, Writing - review & editing. **Vincent Courdavault:** Conceptualization, Funding acquisition, Supervision, Writing - original draft, Writing - review & editing.

## Declaration of Competing Interest

The authors declare that they have no known competing financial interests or personal relationships that could have appeared to influence the work reported in this paper.
